# Ameliorating and refining islet organoids to illuminate treatment and pathogenesis of diabetes mellitus

**DOI:** 10.1186/s13287-024-03780-7

**Published:** 2024-06-27

**Authors:** Yushan Li, Meiqi Xu, Jiali Chen, Jiansong Huang, Jiaying Cao, Huajing Chen, Jiayi Zhang, Yukun Luo, Yazhuo Wang, Jia Sun

**Affiliations:** 1 Department of Endocrinology, Zhujiang Hospital, The Second School of Clinical Medicine, Southern Medical University, Guangzhou, China; 2 Department of Biomedical Engineering, Shanxi Medical University, Taiyuan, Shanxi 030001 China; 3 Nanfang Hospital, The First School of Clinical Medicine, Southern Medical University, Guangzhou, China; 4 Tsinghua-Peking Center for Life Sciences, School of Basic Medical Sciences, Tsinghua University, Beijing, China

**Keywords:** Organoid, Diabetes, Stem cells, 3D culture, Organ transplantation

## Abstract

Diabetes mellitus, a significant global public health challenge, severely impacts human health worldwide. The organoid, an innovative in vitro three-dimensional (3D) culture model, closely mimics tissues or organs in vivo. Insulin-secreting islet organoid, derived from stem cells induced in vitro with 3D structures, has emerged as a potential alternative for islet transplantation and as a possible disease model that mirrors the human body’s in vivo environment, eliminating species difference. This technology has gained considerable attention for its potential in diabetes treatment. Despite advances, the process of stem cell differentiation into islet organoid and its cultivation demonstrates deficiencies, prompting ongoing efforts to develop more efficient differentiation protocols and 3D biomimetic materials. At present, the constructed islet organoid exhibit limitations in their composition, structure, and functionality when compared to natural islets. Consequently, further research is imperative to achieve a multi-tissue system composition and improved insulin secretion functionality in islet organoid, while addressing transplantation-related safety concerns, such as tumorigenicity, immune rejection, infection, and thrombosis. This review delves into the methodologies and strategies for constructing the islet organoid, its application in diabetes treatment, and the pivotal scientific challenges within organoid research, offering fresh perspectives for a deeper understanding of diabetes pathogenesis and the development of therapeutic interventions.

## Introduction

Diabetes mellitus, characterized by hyperglycemia, is a metabolic disease affecting approximately 400 million individuals globally. Chronic inadequate glycemic control increases the risk of microvascular and macrovascular complications, leading to chronic damage and dysfunction across multiple organs [1]. Type 1 diabetes mellitus (T1DM), distinguished by an absolute deficiency of insulin due to T-cell-mediated autoimmune destruction of pancreatic β-cells [2], necessitates lifelong reliance on exogenous insulin injections. While this treatment can control the rise of blood sugar, it fails to maintain blood glucose levels within a normal physiological range, resulting in complications arising from severe fluctuations in blood glucose. Allogeneic islet transplantation offers an effective remedy for Type 1 diabetes, yet its application is limited by a shortage of islet donors and the challenges of allogeneic immune rejection [3]. Type 2 diabetes manifests relative insulin deficiency due to impaired insulin action; its specific causes and pathogenic mechanisms are complex [4]. It can be managed through lifestyle modifications, insulin, and pharmacotherapy [5]. Monogenic diabetes mellitus, arising from mutations in specific genes that disrupt pancreatic islet function, is characterized by early onset and closely related to genetic factors. Advances in gene sequencing technologies have significantly enhanced the diagnosis and treatment of specific monogenic diabetes subtypes, offering more effective targeted therapies. However, research into the specific mechanisms and treatments for diabetes mellitus caused by various genetic mutations remains imperative [6].

Organoids, three-dimensional (3D) tissue constructed from embryonic stem cells (ESCs), induced pluripotent stem cells (iPSCs), adult stem cells (ASCs), pancreatic progenitor cells (PP cells), or partially differentiated cells using in vitro culture techniques [7–9], possess self-renewal and self-assembly capabilities. Under suitable microenvironmental conditions, they can replicate the structure and function of their tissue of origin, making them viable substitutes for tissue or organs in experimental studies in developmental biology, drug screening, disease modeling, and personalized medicine [10–12]. Additionally, they serve as potential organ sources for clinical transplantation, harboring immense potential for diabetes treatment. Although research on islet organoids has commenced, and some progress has been made in some fields, challenges remain, such as incomplete cell type composition, uncontrollable size, shape heterogeneity, lack of a vascular system, and incomplete endocrine function [13].

This paper reviews the progress of islet organoid research in the field of diabetes. Initially, it outlines numerous viable strategies for inducing differentiation and maturation of various source cells during the construction of islet organoids, and introduces current techniques for 3D culture. Thereafter, we review the application of islet organoid across multiple areas of diabetes pathogenesis and treatment research. Lastly, we address a range of challenges encountered in the construction and application of islet organoid, spotlighting existing resolution strategies and unresolved issues.

## Construction of islet organoid

The process of constructing islet organoid typically encompasses three critical steps:

(1) Selection and acquisition of source cells, and induction of these cells to differentiate or transdifferentiate into islet cells. (2) Cultivation of functional islets with 3D structures. (3) Promotion of the morphological and functional maturation of the organoids.

### Induction of cell differentiation

**Fig. 1:**
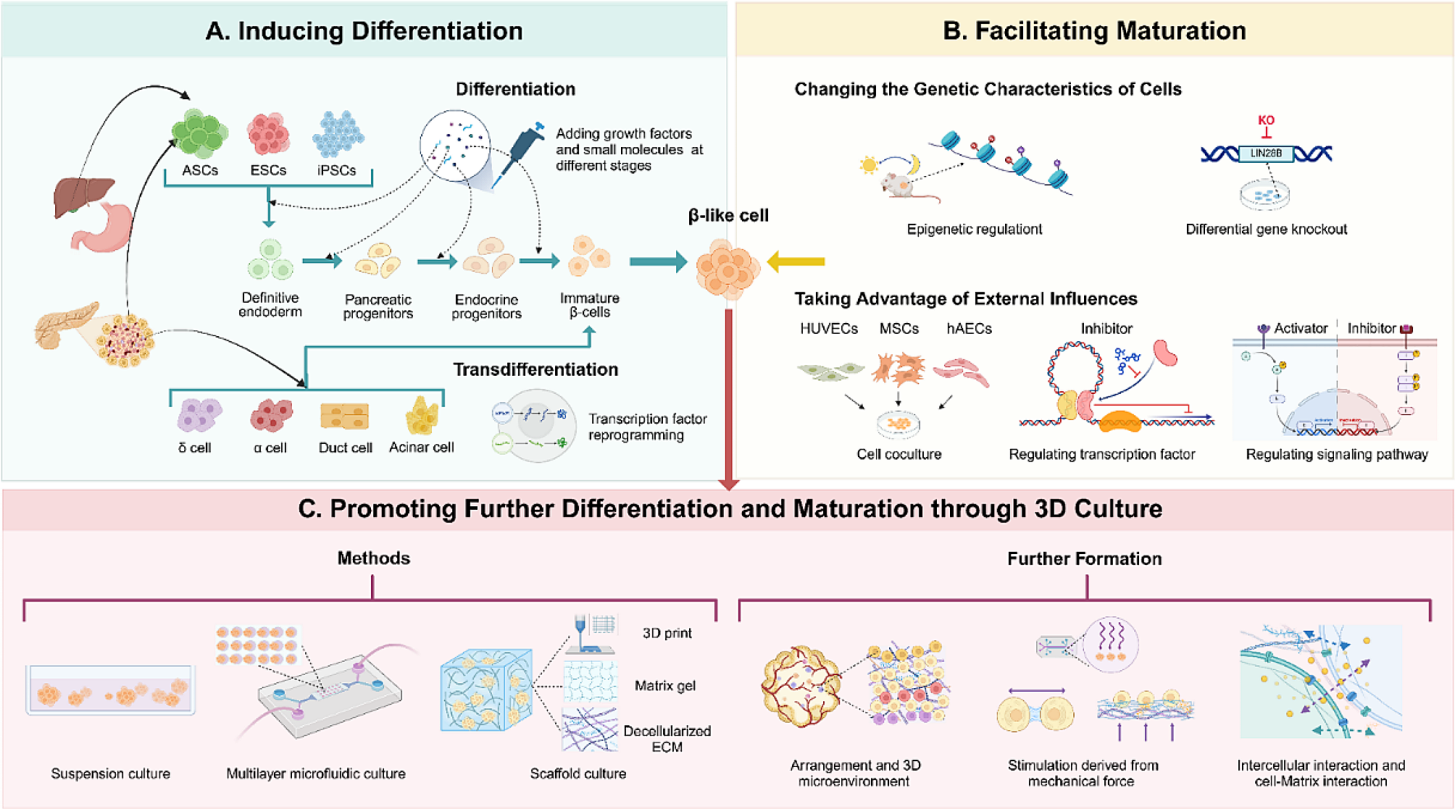
Process and strategies for islet organoid construction. **A**. **Inducing differentiation of cells**. For stem cells derived from different tissue sources, differentiation is induced by applying specific small molecule formula in the culture medium at various stages of cell development. For cells of both the endocrine and exocrine parts of the pancreas, reprogramming technologies are needed to control transcription factors for inducing cell transdifferentiation. **B. Facilitating maturation of differentiated cells.** Islets induced in vitro and implanted in mice, acquire continuous chromatin changes and exhibit more mature functions, such as rhythmic insulin responses, after the establishment of the mice’s diurnal fasting/feeding cycle. Knocking out genes that differ between sc-β cells and more mature natural β cells in humans can also promote maturation. Other strategies to enhance maturation include: co-culturing with auxiliary cells, adding specific molecular inhibitors or activators to regulate transcription factors or signaling pathways related to cell maturation. **C. Promoting further differentiation and maturation through 3D culture.** Compared to 2D culture systems, 3D culture systems better simulate the in vivo environment of cell survival. Common 3D culture methods include: suspension culture, multi-layered microfluidic culture, and scaffold culture. 3D culture systems facilitate the formation of cell arrangement and microenvironments, mechanical stimulation, cell-cell interaction and cell-matrix interaction, thereby further promoting cell differentiation and maturation

The cell sources employed in constructing islet organoid primarily include three types: human pluripotent stem cells (hPSCs), non-pancreatic ASCs [14], and pancreatic tissue-derived cells. These cells are exposed to specific growth factors and signaling molecules in specific doses and sequences to induce their differentiation and maturation [15], as well as the formation of construction, ultimately yielding islet organoid capable of insulin secretion. **(**Fig. 1, **Created with BioRender.com)**.

#### hPSCs

hPSCs, including human embryonic stem cells (hESCs) and human induced pluripotent stem cells (hiPSCs), possess remarkable capabilities for infinite self-renewal and differentiation into all cell types of the three germ layers (namely the endoderm, mesoderm, and ectoderm). This unique functionality renders them ideal candidates for pancreatic islet organ tissue engineering. Based on the elucidated mechanisms of human pancreatic islet development, numerous studies have formulated growth factors and small molecules to modulate key signaling pathways, directing the differentiation of hPSCs toward β-cells [13] (Table 1).

**Table 1:** Function and application of small molecules in the construction of islet organoids

Small molecule	Function	Stages of adding	Application	Stem cell source	Reference
Betacellulin	Epidermal growth factor	PP2 → EN PP1 → PP2	Promotes β-cell growth and differentiation	ESCs、 iPSCs	[32, 116]
T3	Thyroid hormone	PP2 → Mature β cell PP2 → Mature β cell	Promotes β-cell differentiation and maturation	ESCs	[19, 32]
ALK5i	ALK5 inhibitor	PP2 → Mature β cell PP2 → Mature β cell PP → EP PP1 → PH EP → β like cell PP → EP	Promotes β-cell differentiation and maturation	ESCs、 iPSCs	[32, 19, 116, 156, 154, 155]
LDN	BMP inhibitor	DE → EP DE → PP EP → β like cell	Promotes β-cell differentiation and maturation	ESCs	[116, 156, 154]
Noggin	BMP inhibitor	PP2 → EP GTE → EP PGT → EP PGT → PP2	Inhibits hepatocyte lineage differentiation	ESCs、 iPSCs	[116, 154, 155, 166]
Vit C	Epigenetic regulator	PGT → EP GTE → PP1 PE → EC	Increases reprogramming efficiency, promotes pancreatic endoderm induction	ESCs	[19, 154, 153]
Exendin-4	GLP-1 peptide analog	EP → β like cell EP → EC EP → EC	Improves glucose tolerance by increasing insulin secretion	ESCs、 iPSCs	[154, 155, 153]
Nicotinamide	Vitamin	EP → EC EP → EC	Improves glucose tolerance by increasing insulin secretion	ESCs、 iPSCs	[155, 153]
TPB	Protein Kinase C activator	PP1 → EP PP2 → EP	Promotes pancreatic endoderm induction	ESCs	[19, 116]
DAPT	γ secretase inhibitor	Immature β-cell → Mature β-cell PP → PH PE → EP	Blocks Notch signaling/supports long-term self-renewal	ESCs、 iPSCs	[19, 156, 153]
IDE1/2	TGF-β pathway activator	iPSC s→ DE ESCs → DE ESCs →DE	Induces formation of definitive endoderm	ESCs、 iPSCs	[32, 19, 157]
Retinoic acid	Cell signaling molecule	PGT → PH PGT → PP2 PGT → PP1 DE → PP DE → PE	Promotes pancreatic endoderm induction	ESCs、 iPSCs	[32, 19, 116, 156, 153]

**DE**, Definitive Endoderm; **GTE**, Gut Tube Endoderm; **PGT**, Primitive Gut Tube; **PE**, Pancreatic Endoderm; **PP1**, Pancreatic Progenitor 1(PDX1+); **PP2**, Pancreatic Progenitor 2(PDX1+, NKX6.1+); **EP**, Endocrine Progenitor; **EC**, Endocrine Cells(INS++, GCG+, SS+); **PH**, Pancreatic Hormone Expressing Cells; **hPSCs**, Human Pluripotent Stem Cells; **ESCs**, Embryonic Stem Cells; **iPSCs**, Induced Pluripotent Stem Cells;

Research into generating functional β-cells began with hESCs. The earliest differentiation protocol utilized was a five-step induction method. However, this protocol yielded endocrine cell clusters containing only 7% insulin-positive cells, and these cells lacked the capability to respond to glucose stimulation [16]. Subsequent studies have continually refined the differentiation induction protocols. However, cells induced by these protocols require several months to fully mature in vivo, and they retain partial differentiation potential, which poses an increased risk of off-target differentiation and tumorigenesis upon transplantation [17, 18]. Currently, more successful protocol for inducing differentiation and insulin secretion function is the seven-stage differentiation method [19]. This protocol introduces vitamin C in the early stages of hESCs differentiation to generate PP cells with low expression of neurogenin3 (NGN3) and its downstream targets pancreatic and duodenal homeobox 1 (PDX1)/NK6 homeobox 1 (NKX6.1) (Stage 4). Specific reagent combinations, including activin receptor-Like kinase 5 (ALK5) inhibitor, bone morphogenetic protein (BMP) receptor inhibitor, and triiodothyronine (T3), are used to further differentiate these progenitor cells, inducing the upregulation of NGN3 and expansion of the cell population, with a significant portion co-expressing PDX1, NKX6.1, neuronal differentiation 1 (NEUROD1), and NK2 homeobox 2 (NKX2.2) (Stage 5). Subsequently, continuous exposure to ALK5 inhibitor, BMP receptor inhibitor, and T3, along with the addition of a Notch inhibitor, generates a cell population among which a considerable proportion of PDX1/NKX6.1/NEUROD1 cells express insulin, but not glucagon or somatostatin (Stage 6). Finally, combining the AXL inhibitor R428 with the ALK5 inhibitor and T3 effectively induces the expression of the β-cell maturity marker V-MAF avian musculoaponeurotic fibrosarcoma oncogene homolog A (MAFA) in PDX1/NKX6.1/NEUROD1 cells (Stage 7). The resultant highly differentiated cells exhibit key characteristics of mature β-cells, including glucose-induced insulin secretion, and rapidly reverse diabetes upon transplantation into mice [19]. However, due to the requirement for human embryonic destruction, the use of hESCs remains controversial both ethically and politically.

hiPSCs are obtained by reprogramming terminally differentiated somatic cells through the introduction of specific transcription factors. Compared to ESCs, hiPSCs have the advantages of fewer ethical problems and a milder immune rejection response. Importantly, the differentiation of hiPSCs into islet organoids also follows developmental stages observed in hESCs protocols. Previous studies have successfully reprogrammed skin fibroblasts from three T1DM patients into hiPSCs, which were then differentiated into β-cells using induction protocols. The resultant cell clusters contained 24% of β-cells co-expressing C-peptide and NKX6.1, along with a small percentage of α-cells expressing glucagon [20]. The therapeutic efficacy of hiPSC-derived islet organoids for diabetes has also been demonstrated in animal models. Single dose intraportal infusion or prerectal subvaginal implantation of hiPSC-derived islets in non-human primates effectively restore endogenous insulin secretion and improve overall glycemic control [21, 22]. However, single-cell transcriptomic analyses have revealed the presence of various proportions of polymorphic cells, enteroendocrine cells, and unidentified cell types within the hiPSC-derived islet organoid tissues. The functional roles and impact on maturity of these non-target cells within the islet organoid tissues remain unclear [23–26]. Despite the significant advantages demonstrated by hPSCs in the bioengineering of islet organoids, their inherent pluripotency presents a dual challenge due to the difficulty in precisely differentiating them into specific cell types at every step of the process. This has raised concerns about potential off-target differentiation and tumorigenesis associated with undifferentiated cells.

#### ASCs

ASCs, distributed across multiple tissues in the human body, possess the ability for multidirectional differentiation, enabling them to produce tissues corresponding to their originating system. They play a crucial role in renewing and repairing damaged or perished cells within mature tissues. Given the tissue-consistent characteristics of ASCs-derived tissues and their ability to avoid tumorigenicity, ASCs are considered as an additional valuable source for organoid culture.

Current researches have revealed the potential of ASCs to transdifferentiate into cell types outside their own germ layer under appropriate conditions. Endodermal ASCs, such as pancreatic ductal progenitor cells, PP cells, hepatic stem cells, and gastric stem cells, can directly differentiate into insulin-producing β-cells [14, 27, 28]. Additionally, cells from the mesodermal layer, such as bone marrow mesenchymal stem cells (BMSCs), adipose tissue stem cells (ADSCs), dental pulp stem cells, and ectodermal cells like amniotic epithelial cells, can also be induced to transdifferentiate into β-cells [29, 30]. However, the efficiency of differentiating ASCs into β cells varies widely across studies, and their germline origin may be the main reason. For example, overexpressing the three key lineage-specific transcription factors—NGN3, PDX1, and MAFA—in endodermal gastric stem cells can generate organoid tissues that secrete insulin. These organoid tissues consist of approximately 70% β-like cells, along with a minority of α, δ, and ε-like cells. Importantly, these organoids generated in vitro exhibit glucose-stimulated insulin secretion (GSIS) functionality within 10 days and maintain a robust hypoglycemic effect over 100 days post-transplantation in diabetic mice. The time required to generate functional islet organoids from human gastric-origin stem cells is relatively short, and the proportion of β-like cells produced is comparable to that reported for hiPSCs, possibly because both β-cells and gastric stem cells originate from the endoderm [27].

In contrast, current protocols for generating islet organoids from mesodermal stromal cells are not yet of clinical significance for large-scale production. The efficiency of transdifferentiating mesodermal stromal cells into endodermal β-cells remains very low, and there is considerable variability across different protocols. This may result in heterogeneous populations of non-functional polyhormonal or immature endocrine cells (EC cells). In various studies, the differentiation efficiency of BMSCs and ADSCs into C-peptide + or insulin + cells is only about 3–5%, significantly lower than the differentiation efficiency of hESCs and hiPSCs (60–80%), posing a significant barrier to their clinical translation [19, 31, 32].

#### Other types of pancreatic cells

Some glucagon-producing α-cells and somatostatin-producing δ-cells in mice convert to insulin-expressing cells after removal of the β-cells, which promotes diabetes recovery. This scheme is to induce transdifferentiation of other islet cells (such as α-cells and δ-cells) through reprogramming by regulating transcription factors to obtain glucose-responsive islet β-cells [28, 33].

α-cells can be a potential source of β-cells. The identities of α-cells in mice are preserved by aristaless-related homeobox (Arx) [34] and DNA methyltransferase 1 (Dnmt1) [35], and the pathways regulating Arx and Dnmt1 is crucial for the transformation of α-cells into β-cells. Insulin-producing cells also might emerge through the spontaneous regeneration of α-cells. Using doxycycline to stimulate expression of Cre recombinase, which in turn inactivated Arx and Dnmt1, researchers subsequently found that within three months of loss of Dnmt1 and Arx, 50-80% of α cells were transformed into progeny cells resembling natural β cells. Moreover, these transformed α-cells exhibited β-cell electrophysiological traits and demonstrated GSIS [28]. Therefore, blocking the expression of these two genes with the use of targeted approaches or controlling their signaling could be a way to promote the transformation of α cells into β cells in islets.

Ductal cells possessing stem cell characteristics is another source of β-cell [36]. Co-expression of Ngn3, Pdx1, and MAFA prompted mouse ductal cells to differentiate into β-like cells, with inhibition of Ngn3 phosphorylation at cyclin-dependent kinase 9 (CDK9)-targeted sites, significantly enhancing β-cell generation efficiency, surpassing other similar reprogramming environments [37]. Nevertheless, for these three factors, expression of only one or two of them in duct organoid prompted the production of other pancreatic hormones rather than insulin [37]. Two types of source cells mentioned above could transform into β-cells under specific circumstances, providing a new strategy for restoring β-cell function in diabetic patients.

Moreover, acinar cells in exocrine pancreas also serve as a source for insulin-positive β-like cells. Introducing lentiviruses expressing activated mitogen-activated protein kinase (MAPK) and signal transducer and activator of transcription 3 (STAT3) into EC cells derived from adult pancreatic tissue activated 50–80% of these transduced cells to express the endocrine-promoting factor Ngn3 in both monolayer and 3D culture systems. Besides, lineage tracing confirmed that the ultimate insulin-producing cells originated from acinar cells [38].

#### Clinical translation and source cells selection

Although islet organoids derived from hPSCs, ASCs have achieved varying degrees of clinical translation, there are significant discrepancies in the outcomes and improvements of these protocols corresponding to different cell sources [39]. This discrepancy may explain the current focus in stem-cell-derived β cells (sc-β cells) and stem cell-derived pancreatic islets (sc-islets) -related clinical trials **(**Table 2**)** on the results and refinements of the induction protocols, such as the differentiation efficiency and maturity of the cells, rather than primarily focusing on the advantages and disadvantages of the different source cells themselves.

**Table 2:** Current clinical trials offer promising insights into the potential future clinical transplantation of islet organoid

Intervention	Details	Anti-immune rejection strategy	Transplantation site	Phase	Enrollment	Status	Trial ID	Reference
VX-880	sc-islets derived from allogeneic human stem cells	Immunosuppressive agents	Liver (Portal vein)	1 and 2	17 (Estimated)	Recruiting	NCT04786262	[49]
VX-264		Channel array device encapsulating SC-islet	Liver (Portal vein)	1 and 2	17 (Estimated)	Recruiting	NCT05791201	[158]
VCTX210	Allogeneic pancreatic endoderm cells (PEC210A/PEC211A)	Gene editing by CRISPR/Cas9 (including but not limited to knocking out the β2-microglobulin gene encoding part of HLA I molecules, and inserting the PD-L1 transgene) PEC-Direct (encapsulating PEC210A/PEC211A without immune protection )	Subcutaneous space	1	7	Completed	NCT05210530	[159]
VCTX211			Subcutaneous space	1	40 (Estimated)	Recruiting	NCT05565248	[160]
VC-01™	Allogeneic pancreatic endoderm cells (PEC-01/PEC-02) derived from hESC	PEC-Encap (encapsulating PEC-01 within a semi-permeable membrane)	Subcutaneous space	1 and 2	19	Terminated, most cells died due to lack of oxygen and nutrients	NCT02239354	[161]
VC-02™		Immunosuppressive agents with PEC-Direct (encapsulating PEC-01/PEC-02 without immune protection)	Subcutaneous space	1 and 2	49	Active, not recruiting	NCT03163511	[162]
Donislecel	Allogeneic islets	Immunosuppressive agents	Liver (Portal vein)	3	30	Approved for marketing	NCT03791567	[163]
Islet transplantation	Allogeneic islet cells	Immunosuppressive agents	Omentum	1 and 2	4	Completed	NCT02821026	[164]
Islet transplantation	Allogeneic islets	Cell Pouch™ (a scaffold made of non-degradable polymers) and immunosuppression (a minimum of three weeks after Cell Pouch implantation)	Abdominal musculature	1 and 2	13 (Estimated)	Recruiting	NCT03513939	[165]

**CRISPR/Cas9**, Clustered Regularly Interspaced Short Palindromic Repeats-Associated protein 9; **HLA**, Human Leukocyte Antigen; **PD-L1**, Programmed Death Ligand 1

For ESCs, despite ethical concerns, accessibility issue, as well as potential immunogenicity which may increase the risk of immune rejection [40], the known source cells in existing sc-β cells and sc-islets clinical trials are hESCs. This preference may be due to their high differentiation potential and their role as the source cells in the earliest functional β-cell production protocol [17, 18]. Their induction and differentiation protocols have undergone significant improvements, producing sc-β cells with better functionality. For iPSCs, although they possess high differentiation potential similar to ASCs and better accessibility, along with the unique advantage of reducing immune rejection due to their derivation from patient autologous cells, their large-scale application remains constrained by challenges associated with reprogramming technology. For instance, the commonly used viral vector methods with high efficiency pose risks of genomic integration and tumorigenesis [41], and are also likely to cause inflammatory responses due to strong immunogenicity [42]; non-viral methods, such as chemical reprogramming [43], although relatively safe, are less efficient and might revert to earlier differentiation states, with long-term effects of small molecules on signaling pathways yet to be clarified. For ASCs with relatively low tumorigenic potential, the main challenge is their low differentiation efficiency. For example, BMSCs which are easily accessible and have low immunogenicity [44], can secrete nutritive factors that promote the survival and functionality of transplanted islets [45]. However, due to their mesodermal origin, which differs from the endodermal origin of pancreatic, their transdifferentiation efficiency is lower compared to gastric stem cells derived from the endoderm [46, 47].

Similarly, the transdifferentiation of non-β pancreatic cells, compared to the differentiation of hPSCs, exhibits a significant gap in conversion efficiency, and the feasibility of ductal cell transdifferentiation is also controversial [48], which make it a secondary option for clinical applications. However, their substantial numbers and the advantage of same developmental origin make related differentiation strategies still valuable for research and improvement.

From the discussion above, it can be concluded that currently, the use of hPSCs as a cell source for generating sc-β cells and sc-islets holds significant advantages for clinical translation. However, off-target differentiation and maturation defects remain unresolved issues. In clinical trials, although fully differentiated sc-islets can be obtained through sorting methods [49], it is uncertain whether these cells are functionally significant in terms of single-hormone-secreting islet cells, their differentiation and maturation may also be influenced by the transplantation device and site. Adjusting the differentiation induction protocols of specific cell sources based on cell examination results obtained from clinical trials can lead to the generation of more mature and functional islet cells. This targeted approach, is currently a major concern in clinical trials, as compared to considering the differences in outcomes based on different cell sources [50].

### 3D culture of islet organoids

Organoids formed based on conventional monolayer culture systems differ significantly from the multicellular components of human islet function and do not possess the complex biological structure of natural islets. Given the crucial role of islet cell structure in endocrine function, novel systems for 3D organoid cultivation that mimic the intrinsic cellular environment—including cell polarization, arrangement, and niche—are pivotal for the efficient differentiation and maturation of β-cells [51]. There are two approaches in cultivating islet organoid: one cultures hPSCs in suspension to utilize their self-organizing ability for generating 3D structure; the other employs constructed scaffolds, on which cells are cultured to facilitate the formation of 3D structure.

Compared to traditional monolayer culture, suspension culture reduces cell-to-cell adhesion and maximizes interactions between cells and the extracellular matrix (ECM). Making use of the inherent plasticity for genetic modification and self-organizing capability of hPSCs and dispersing these cells in a static suspension for culture, during which the cells were able to recombine to form a number of embryoid bodies (EBs) with appropriate 3D structures and insulin release patterns. Dynamic suspension culture is one strategy that can more efficiently control the formation of EBs. This cultivation strategy comprises three main phases: controlled aggregation of EBs, induction of pancreatic differentiation, and maturation of islet organoids [52]. It used an organ-on-a-chip platform containing a multilayer microfluidic device that allows perfusion culture in a single device with precise control of mechanical flow of fluid, biochemical signals, and cell-cell interactions. The culture device had micropores and channels to form a perfusion system that can supply nutrients for a long time. The suspension was injected into the upper layer of the device, with isolated hiPSC cells being evenly dispersed in the channels and micropores. During this dynamic perfusion culture, hPSCs formed EBs through their self-organizing ability, forming the original form of islet organoid. Then, as mentioned above, different chemokine formulas were used in the system to induce hPSC differentiation towards insulin-secreting cells. These chemokines can be controllably added to the system via pumping devices located on the upper and lower layers of the organ-on-a-chip platform [52]. The microenvironment encompassing derived cells is particularly important for the cultivation of islet organoid. In this device, under the condition of perfusion culture, the expression of genes and proteins related to β-cell maturation was enhanced, with an increased insulin secretion; differential osmotic pressures generated between the microwell arrays influenced the growth of the organoids, and the primitive morphology of the organoids stayed spherical and had smooth edges [52].

Constructing scaffolds to promote the formation of cell clusters is another cultivation strategy. Natural matrices, such as the ECM used as scaffolds, enhance cell-matrix interactions and the development of 3D structures. Another approach is to provide a unique biomimetic 3D microenvironment by applying decellularization techniques to porcine pancreas, which reserves ECM components as well as the 3D structure [53]. Traditional collagen scaffolds also provide an ideal ecological environment, but these scaffolds tend to collapse during the cell differentiation process. Incorporating matrix gel into collagen scaffolds may improve the mechanical strength of them [54]. In addition, constructed “Amica gel” platform, where definitive endoderm (DE) and PP cells derived from hESCs were seeded onto plates coated with Amica gel with a mixture of endothelial cells (ECs) and cultured for 14 h. Owing to the unique surface characteristics of Amica gel, the PP cells and ECs underwent self-aggregation and spontaneous integration to form heterogeneous spheroids [55]. Subsequently, differentiation factors were added to the spheroids to induce further differentiation [55]. For the culture of organoid, in addition to employing reasonable signaling molecule formulas to guide cell-specific differentiation, rational use of cell-matrix interactions and creation of specialized 3D scaffolds to promote EC cells differentiation also should not be ignored.

### Strategies for the maturation of islet organoid

A mature islet is an endocrine organ that contains various EC cells, ECM, and a complete vascular system, capable of fully functioning in insulin secretion. Following the aforementioned induction and cultivation protocols, islet organoids still have two major limitations compared to natural islets: immature endocrine function and a lack of perfusable vasculature. Therefore, it is necessary to further explore tissue engineering techniques and gene editing methods to successfully generate functional islet tissues for the treatment of diabetes.

#### Auxiliary cells for islet maturation

In addition to EC cells of islets, non-endocrine cells such as ECs and mesenchymal cells also play a supportive role in the survival and functioning of the islets. Co-culturing induced differentiated cells with other cell types to leverage their unique properties is an indispensable part of constructing mature islet organoids.

Islets have a rich vascular supply that provides sufficient oxygen and nutrients to the human islets and can precisely regulate blood glucose. ECs, which constitute the vascular network, not only actively regulate vascular permeability and promote vascularization of islet tissues, but also modulate the expression and movement of a number of important immune mediators. In addition, ECs produce a variety of factors, such as connective tissue growth factor, BMP-2, and BMP-4, which increase the insulin expression in adherent cultures of hESCs-derived PP cells and mediate islet-specific maturation. Human umbilical vein endothelial cells (HUVECs) or ECs mixed with human PP cells promote self-condensation, maturation and vascularization of islet organoid [55]. In addition to exogenous addition of ECs, EGF also induces angiogenesis in human islet tissues [56]. Recently, angiopoietin-2 was shown to induce widespread dissemination of vascular endothelial cadherin (VE-cadherin) + ECs and neuron-glial antigen 2 (NG2) + pericytes within islet tissues. In addition, angiopoietin-2 partially complements the paracrine stimulation of islet cells by ECs in vivo.

Similar to ECs, mesenchymal stromal cells (MSCs) are involved in the construction of organoid tissues. First, MSCs can trigger the initiation of self-condensation, a traction force generated by the actin cytoskeletal axis that leads to directional violent movements of the cellular collective [57, 58]. By co-culturing endocrine mouse insulinoma 6 cells with HUVECs and MSCs, self-organized islet organoid tissues were constructed, which restored body weight and blood glucose levels to normal in diabetic mice after transplantation [59]. Subsequently, hESCs-oriented PP cells, MSCs, and ECs were seeded on Matrigel at optimized cell ratios to self-organize islet tissues [60]. In addition to triggering self-organization, ECs interactions were supported by MSCs in the intramuscular human pancreatic islet transplantation model. Human pancreatic islet ECs migrate from the transplantation center to the surrounding tissue and form chimeric vessels with recipient ECs [61]. In addition, MSCs support encapsulated mouse islets in a hydrogel environment by exerting paracrine functions, such as the release of trophic factors such as hepatocyte growth factor (HGF) and transforming growth factor beta (TGF-β) by bone marrow-derived rat MSCs, and show an increase in insulin secretion in response to glucose challenge [62].

#### Genetic regulation of islet maturation

In addition to adding specific cellular components to the system, regulation in molecular level is also a strategy to promote cell maturation. Employing multi-omics analysis enables the identification of differences between islet organoid and natural pancreatic islets, for example, mRNA seq of sc-β cells and human islet cells revealed that sc-β cells have elevated levels of lin-28 homolog B (LIN28B), which may impede the maturation of pancreatic islet-like organs. Knockdown of LIN28B significantly improved GSIS in these islet organoids [63]. Furthermore, epigenetic analysis of hPSC differentiation revealed that differentiated pancreatic islets acquired rhythmic insulin responses and sustained chromatin changes by establishing a circadian fasting/feeding cycle in mice. This evidence suggests that adjustments to the circadian rhythm alter the epigenomic dynamics of islet organoids, facilitating their function advancement [64]. Other strategies, such as changing the activity of non-classical wingless/integrated (WNT) signaling, can also be used to regulate the function of induced differentiated organoids [65]. Existing studies have confirmed that the activation of non-classical WNT signaling via wingless-type MMTV integration site family, member 4(WNT4) can enhance β-cell maturation, improving the secretory capabilities of islet organoids cultured in vitro [66].

The mouse model mentioned above serves as a useful tool for studying the developmental process of human islets and strategies to enhance their maturation [67]. However, differences exist between mice and human in the transcription factor regulatory mechanisms governing islet development, for instance, v-maf avian musculoaponeurotic fibrosarcoma oncogene homolog B (MAFB)—a critical transcription factor modulating β-cell maturity within the mouse pancreas—vanishes after reaching adulthood, whereas, it persists during human pancreatic development into adulthood [68].The application of islet organoids to the study of pancreatic development and maturation processes has unveiled similar regulatory mechanisms that differ across species. For example, despite early human epigenomic research indicating the transcriptional repressor RE1-silencing transcription factor (REST) as an inhibitor of embryonic pancreatic endocrine formation, observation that pancreatic Rest knockout mice displayed no anomalies in the number of EC cells. With regard to this difference, it was observed that inhibiting REST in organoids derived from adult pancreatic ducts with X5050 treatment led to the activation of β-cell-specific genes though the number of pancreatic EC cells produced after this treatment was minimal, suggesting that regulating negative transcription factors can enhance the maturation of human β-cells [69].

## Application of islet organoid

### Organoid / beta cell replacement therapy

**Fig. 2:**
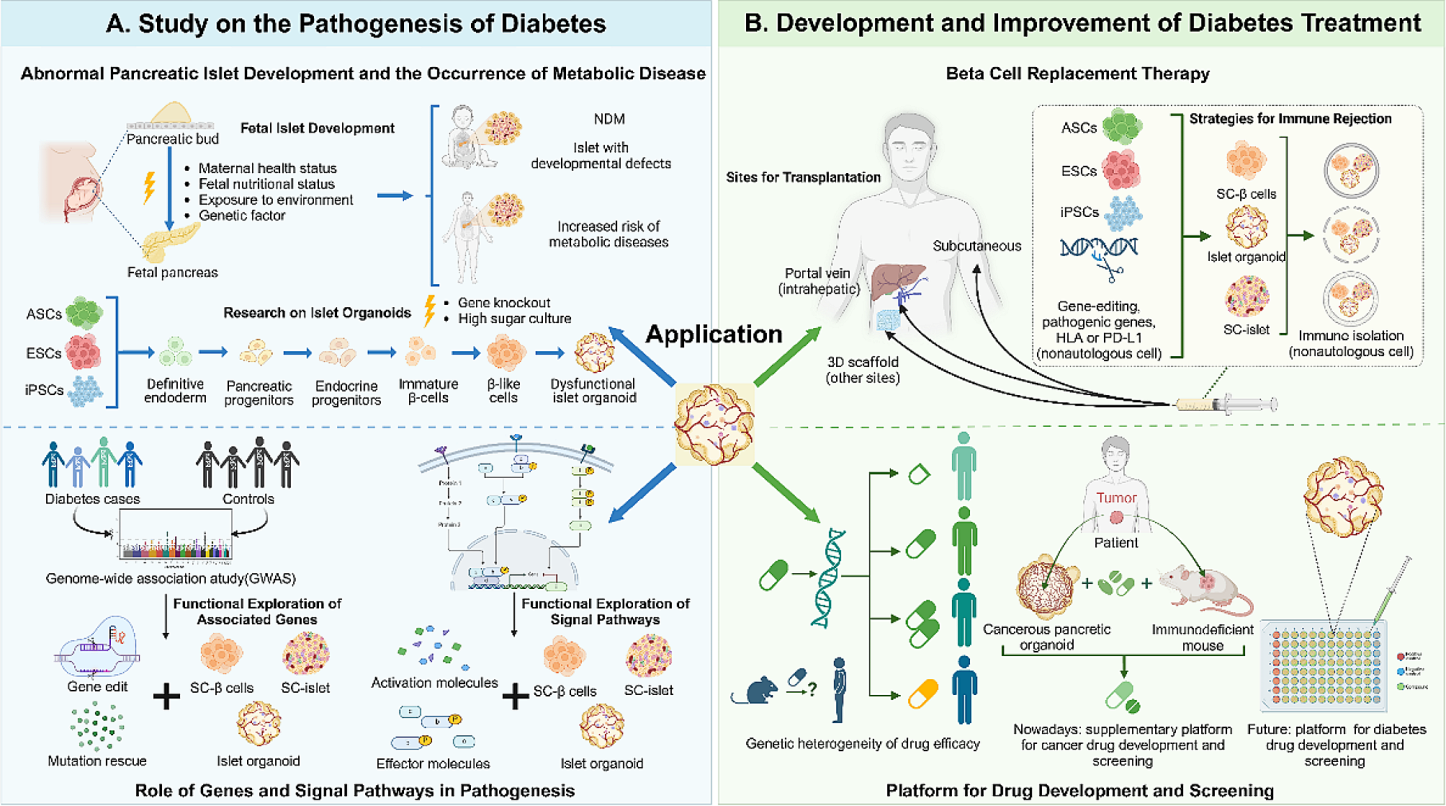
Applications of islet organoid in pathogenesis research and treatment improvement of diabetes. **A. Study on the pathogenesis of diabetes**. During fetal development, effects such as maternal health status, nutritional supply, environmental exposure, and genetic factors can contribute to abnormal pancreatic islet development, leading to neonatal diabetes or increased risk of metabolic diseases in adulthood. The process of in vitro induction of cell differentiation and maturation to construct islet organoids can simulate islet development and explore the specific roles of these factors in mechanisms of disease onset and increased disease risk **(upper left)**. GWAS and various genetic methods have identified a large number of genes associated with the onset of diabetes. Through gene editing and gene mutation rescue techniques applied to patient-derived cells and the construction of sc-β cells, sc-islets, or islet organoids, it is possible to eliminate species genetic differences and individual genetic background heterogeneity, clarifying the pathogenic role of genes. Similarly, by adding activating molecules or effector molecules to islet organoids, disease-related signaling pathways can be regulated, and their involvement in disease mechanisms clarified **(lower left)**. **B. Development and improvement of diabetes treatment.** Islet transplantation is an effective treatment for diabetes. Using islet organoids as a source for transplantation offers the advantage of low risk of immune rejection (derived from the patient’s own cells or through transplantation rejection-related gene editing), and their rich source also facilitates exploration of new transplantation sites and the development of encapsulation materials **(top right)**. The existence of pharmacological heterogeneity among species and individuals makes organoids of significant value in the field of tumor drug screening and development, where they are now widely applied. Currently, the efficacy of several islet organoid-based diabetes drug screening platforms has been preliminarily verified, and their potential value of high efficiency, convenience and precision medicine is expected to be further exploited in the future **(bottom right)**

For T1DM patients who are not indicated for exogenous insulin therapy, beta-cell replacement therapy—including pancreas transplantation, islet transplantation, and beta-cell transplantation—presents an alternative feasible option. Currently, allogeneic islet transplantation faces challenge of lacking cadaveric donor islets. Compared with allogeneic islet transplantation, islet organoid technology has the potential to produce islet cells at a larger scale, facilitating the exploration of alternative transplantation sites. Additionally, it can also address the problem of immune-mediated damage by incorporating immunomodulatory cells or utilizing patient-derived autologous stem cells as the source [70]. (Fig. 2, **Created with BioRender.com)**.

Viacyte’s Phase I/II clinical study demonstrated that PP cells, derived from directed differentiation of ESCs in vitro and encapsulated in a non-immunoprotected biocompatible capsule, were subcutaneously transplanted into patients with T1DM [71]. Clinical trial results from the initial cohort of 15 patients from a single trial site indicated that the PEC-01s could survive for up to 26 weeks post-implantation and differentiate into insulin-secreting cells responsive to glucose. However, due to the inefficient further differentiation of PP cells into β-cells in vivo, this therapy has not yet been able to restore blood glucose levels to healthy levels. Additionally, Vertex Pharmaceuticals has also conducted clinical trials of transplant therapies for T1DM using regenerated islet tissue derived from ESCs [72]. In the Phase I/II clinical trial, two patients received a half-target dose of transplant therapy, and 15 patients received a full dose. Currently, one patient who received a half-target dose achieved and maintained insulin independence 27 days post-treatment. However, constructing islet tissue from pluripotent stem cells (PSCs) faces significant challenges due to the highly complex intermediate differentiation processes, particularly concerning the purity of target cells and the retention of off-target cells, especially the risks associated with in vivo tumorigenesis from residual undifferentiated cells within the system. In recent years, the search for new types of stem cells downstream of PSCs, which possess specific differentiation potential without the risk of in vivo tumorigenesis, has become a research focus.

Recently, the first autologous islet organoid transplant trial was conducted in humans [73]. Researchers reprogrammed the patient’s own peripheral blood mononuclear cells (PBMCs) into iPSCs, subsequently establishing endoderm stem cells (EnSCs) with specific differentiation potential toward the endodermal lineage. Using EnSCs as the seed cells, islet tissues were reconstructed in vitro. A patient with pancreatic islet dysfunction due to type 2 diabetes mellitus (T2DM) received this iPSC-derived islet organoid transplant. After transplantation, the patient’s glycemic control progressively improved, with blood glucose fluctuations returning to above 99% stability from week 32, and both severe hyperglycemia (> 13.9 mM) and severe hypoglycemia (< 3.9 mM) completely resolved within two weeks after operation. The variability of preprandial and postprandial glucose levels decreased from 5.50 mM before surgery to 1.60 mM at week 32, achieving insulin independence for up to 33 months. EnSCs based islets (E-islets), possess more specific differentiation potential, leading to a lower risk of tumorigenesis in vivo. Due to the endodermal specificity of EnSCs and their closer developmental proximity to the pancreatic lineage, they are suitable for efficient large-scale production of islets. Moreover, selecting T2DM rather than T1DM as the indication not only facilitates the assessment of E-islets in vivo safety and efficacy under conditions that avoid the complications of autoimmune rejection, but also expands the clinical application scenarios for sc-islets. However, this study cannot entirely exclude the possibility that residual endogenous islets benefited from the surgery and experienced function improvement. Therefore, increasing the sample size and conducting more trials in T1DM patients with complete loss of islet β-cells will help clarify the role of E-islets in achieving glycemic targets. In the future, longer follow-up periods are anticipated to assess effectiveness and safety.

The clinical implementation of islet organoid transplantation therapy requires the development of more strategies to address and improve existing issues. The lack of production standard with good reproducibility and safety hinders analyzing and interpreting the heterogeneity of results in clinical transplantation studies with small sample sizes [71]. More similar studies are needed to develop large-scale production processes that comply with Good Manufacturing Practices (GMP) and have precise quality control, facilitating the advancement of islet organoids for the clinical treatment of T1DM [74]. Cancer-related mutations emerging in iPSCs after the reprogramming of patient fibroblasts directly halted the clinical study of iPSC-induced cell transplantation therapy [75]. This suggests underscores the necessity for comprehensive analyses, such as large-scale studies on hiPSC mutations, to elucidate the impacts of these mutations on cell functions and pathologies. It is also critical to conduct cautious safety assessments before transplantation to mitigate the potential risks associated with stem cell-derived cell transplantation therapies [76]. In the future, gene editing could not only correct mutations in patients but can also enhance function of islet, enabling personalized treatment for individuals.

### Drug screening, development and precision therapy

Diabetic patients exhibit genetic heterogeneity in their response to drugs, therefore, tailored drug screening and precision therapy for each patient are particularly important. Organoids developed from individual patient cells, which are easily scalable and reproducible, have been used for screening personalized therapeutic drugs in cancer patients [77]. Extending the application of organoids in drug screening development from tumors to diabetes holds considerable promise. The feasibility of using sc-β cells and islet organoids to screen for potential therapeutic drugs related to specific gene mutations in diabetes has been validated in multiple studies. Through high-content chemical screening of hESCs-derived β-cells carrying T2DM susceptibility gene mutations identified by genome wide association study (GWAS), researchers identified a candidate drug T5224 which could rescue glucose secretion defects in β-cells due to CDKAL1 mutations by inhibiting FBJ murine osteosarcoma viral oncogene homolog (FOS)/ Jun proto-oncogene (JUN) pathway [78]. In addition to restoring β-cell function, maintaining normal β-cell numbers either by inhibiting their death or promoting the proliferation of residual β-cell has also been a focus of drug development. For example, a high-content chemical screening on β-like cells derived from hESCs with GLI-similar 3 (GLIS3) -/- mutations (associated with T1DM, T2DM, and neonatal diabetes mellitus (NDM) was performed using existing clinical trial drugs. It was discovered that the drug candidate galunisertib rescued β-cell death associated with GLIS3 mutations, which is expected to be applied to the precision treatment of diabetes related to GLIS3 mutations [79]. In another study, research based on human islet organoid models confirmed that a small molecule inhibitor of the dual-specificity tyrosine phosphorylation regulated kinase 1 A (DYRK1A) kinase effectively promotes β-cell proliferation within organoids, enhancing their long-term insulin secretion and balanced glucagon levels maintenance, which offers promising prospects for islet repair treatment in diabetic patients [80].

However, the above drug screening platforms based on sc-β cells or islet organoids have the problem of lacking the integrated environment of other organs related to diabetes pathogenesis. Application of organ-on-a-chip technology to combine islet organoids with other organoids for drug screening is a possible solution. Combined liver-islet organoids system was built through 3D co-culture of 30 days after using hiPSC to construct liver and islet organoids separately [81]. By testing the response of the combined organoid system to metformin therapy, it was confirmed that this system has a potential to screen for novel drugs of T2DM. In addition, combined islet-intestinal organoids chip comprising pancreatic β-cells and small intestinal L-cells was constructed by taking advantage of the highly controllable perfusion culture in organ chips, which allows for automatic, real-time assessment of islet function. This setup was validated for its function in assessing the dynamics of glucagon-like peptide-1 (GLP-1) and insulin in a glucose-dependent environment, offering a novel platform for screening GLP-1 analogs and stimulants for diabetes therapy [82]. However, these platforms for applying islet organoids for the development and screening of diabetes therapeutic drugs still require further improvement. The materials used in these screening systems, such as the polydimethylsiloxane (PDMS) in organ-on-a-chip setups, absorbs hydrophobic molecules from cell culture matrices and devices, potentially impacting drug concentration and pharmacological activity [83]; Matrigel, which is widely used for organoid construction, might affect drug penetration, thus interfering with the outcomes of drug screening [84]. In the future, the combination of islet organoid technology and organ-on-a-chip technology with multiple interconnected organs will preserve the influence of various metabolically relevant organ interactions on disease onset and progression, offering a superior screening platform for drug development in multisystem diseases such as diabetes [85].

### Research on pathogenesis of diabetes

Gene mutations are a significant cause of various types of diabetes. With the advancement of genetics technology and the application of techniques such as candidate gene strategy, homozygosity mapping, next-generation sequencing, and GWAS, more than 40 subtypes of monogenic diabetes associated with mutations in genes, such as glucokinase (GCK) and hepatocyte nuclear factor 1 alpha (HNF1A) have been identified so far. Additionally, the onset of T2DM in some patients is influenced by both monogenic diabetes genes and other susceptibility genes, and the number of risk genes that have been identified to be highly correlated with the pathogenesis of T1DM and T2DM is also continually growing [86–88].

At the cellular level, studies based on human sc-β cells or islet models can further clarify the specific pathogenic mechanisms of pathogenic genes, functional genes, and diabetes-related genes. In the case of tyrosine kinase 2 (TYK2), a risk gene associated with T1DM outside the human leukocyte antigen (HLA) region, researchers knocked it out in hiPSC and induced the generation of mature sc-islets, confirming that TYK2 deletion suppressed the processing and presentation of interferon alpha (IFNα)-induced antigens (such as HLA class I and class II molecules), with the potential to inhibit the progression of T1DM [89]. In another study, pancreatic β-like cells derived from hESCs with a deletion of the T2DM-associated gene CDK5 regulatory subunit associated protein 1-like 1(CDKAL1), obtained through induced differentiation, expressed insulin but exhibited functional impairments and hypersensitivity to glucolipotoxicity. However, forced expression of metallothionein 1E (MT1E), a member of the metallothionein (MT) family significantly down-regulated in cells deficient in CDKAL1, was able to rescue the defects of pancreatic β-like cells. This rescue effect may be associated with the role of MT1E in alleviating endoplasmic reticulum (ER) stress [90]. For Wolfram syndrome(WFS), which includes insulin-dependent diabetes mellitus and caused by WFS1 mutations, β-cells derived from patients’ iPSCs have reduced insulin levels along with increased levels of ER stress molecules. Whereas, treatment with the molecular chaperone 4-phenylbutyric acid, which assists in protein folding and transport, restores insulin levels of iPSC-derived β-cells from patients, suggesting that WFS1 has an inhibitory effect on ER stress damage in β-cells, and the absence of this effect may be a direct cause of diabetes in patients with WFS [91].

At the organ level, compared to mouse model, organoids can better maintain the functional and physiological traits of patient-derived cells [92] and can overcome the defects of lacking species similarity and genetic diversity in model mice [93], thus facilitating the elucidation of gene pathogenic roles in humans that differ from those in mice. For example, pancreas and intestinal organoids constructed with PSCs from patients carrying neurogenin 3 (NEUROG3)mutations clarified that mutations in human NEUROG3 consistently result in the absence of intestinal enteroendocrine cells (EEC), but some of the mutant subtypes have preserved pancreatic endocrine function and do not exhibit severe diabetes, distinctly diverging from mouse NEUROG3 mutations, which invariably lead to a loss of pancreatic endocrine function [94]. Additionally, the application of gene editing technologies such as zinc finger nuclease (ZFN), transcription activator-like effector nuclease (TALEN), and Clustered Regularly Interspaced Short Palindromic Repeats-Associated Protein 9 (CRISPR/Cas9) to organoids has an advantage over their use in mice, including higher efficiency, excellent reproducibility, and rapid functional validation [93]. It also allows for more precise and controllable introduction and selection of mutations [95], eliminating genetic heterogeneity of patients and clarifying whether diabetes is solely caused by a specific mutation. To investigate the specific pathogenic role of hepatocyte nuclear factor 1 alpha p291 frameshift insertion C (HNF1αp291fsinsC) truncation, the most common pathogenic mutation in maturity-onset diabetes of the young type 3 (MODY3), researchers constructed islet organoids using hiPSC derived from patients with MODY3. They clarified that this pathogenic mutation diminishes PP cell number and hinders β-cell differentiation by disrupting interaction between HNF1αp291fsinsC. The study also found varying degrees of partial rescue related to differences in hepatocyte nuclear factor 1 beta (HNF1β) expression among patients, offering a potential explanation for why MODY3 patients with the same genetic mutation exhibit differing clinical manifestations. Moreover, by using CRISPR/Cas9 to introduce the HNF1αp291fsinsC mutation into HNF1α WT iPSC lines, researchers eliminated the influence of the heterogeneous genetic backgrounds, confirming that the same disease phenotypes observed in HNF1α MODY3 patients were solely caused by the frameshift mutation in HNF1α [96].

Islet development and β-cell proliferation and maturation are closely associated with certain signaling pathways [97]. Abnormalities in these signaling pathways may lead to diabetes. Partial single-nucleotide polymorphisms (SNPs) in transcription factor 7 like 2 (TCF7L2) gene, corresponding to TCF7L2, a key transcription factor in the WNT signaling pathway, are associated with an increased risk of T2DM. Mutation of TCF7L2 is associated with abnormalities in insulin secretion and islet cell function, indicating that the WNT signaling pathway may be involved in the pathogenesis of T2DM [98]. The inhibition of the WNT signaling pathway by increased accumulation of nuclear forkhead box O transcription factors (FOXOs), associated with the activation of the reactive oxygen species (ROS) and c-Jun N-terminal kinase (JNK) signaling pathways during aging, may explain the chronic pathogenic course and age-dependent pathogenesis of [99].In addition to the above signaling pathways related to β-cell function and senescence, those regulating β-cell proliferation may also play a role in the onset of diabetes mellitus. To compensate for the defects of non-human models and to explore the genetic regulation mechanism of human β-cell replication, sc-β cells replication was induced by using the yes-associated protein (YAP)(Hippo pathway effector) overexpression system, and single-cell sequencing demonstrated the up-regulation of the leukemia inhibitory factor (LIF). Further adding LIF to the suspension culture of the sc-β cell clusters and confirmed that activation of the LIF-related pathway promotes β cell proliferation. This indicates that mutations in the LIF gene, known to be associated with T2DM, may actually contribute to the development of diabetes through the effects of the LIF-related pathway [100]. Compared to sc-β cell model cultured in vitro, the islet organoid model, which is closer to the human in vivo environment, may provide further insights into the relationship between signaling pathways and islet cell function, as well as their role in the development of diabetes, under conditions that support interactions between multiple cell types.

### Research on the mechanism of other metabolic diseases

Numerous factors during fetal development, including nutritional supply, maternal health status, genetic factors, and exposure to environmental pollutants, are closely linked to the development of metabolic diseases. Although these factors not directly cause metabolic diseases, they heighten the likelihood of developing metabolic diseases in adulthood.

Intrauterine growth retardation (IUGR), resulting from insufficient nutrition and oxygen supply, leads to insulin resistance and defective β-cell secretion after birth. This condition progressively evolves into a declining trend in total β-cell mass in adulthood, consequently increasing the risk of developing metabolic diseases such as obesity, fatty liver, and diabetes in adulthood [101, 102]. Cultivating hESCs in a high-glucose environment, which simulated the impact of maternal diabetes on fetal development, resulted in a diminished potential for pancreatic lineage differentiation in hESCs and over 2000 differentially methylated cytosine-phosphate-guanine (CpG) sites similar to the abnormal pancreatic DNA methylation observed in T2DM patients. This indicates that maternal diabetes might increase the offspring’s risk for T2DM by impairing fetal pancreatic development through epigenetic regulation alteration [103].

Genetic factors, such as mutations in genes involved in islet development and β-cell function formation, can also lead to abnormal islet development, reduced β-cell mass or dysfunction of β-cell, which can result in hereditary diabetes [104, 105]. Juxtaposed with another zinc finger gene 1(Jazf1) is a genetic susceptibility gene associated with T2DM, resulting in a decrease in the expression of genes related to β-cell differentiation in β-cells derived from Jazf1-knockout iPSCs as well as notably lower levels of insulin and C-peptide in the differentiated insulin precursor cells (indicating impaired β-cell differentiation) [106]. Patients with Mitchell-Riley Syndrome, which includes NDM, often exhibit mutations in the regulatory factor x 6 (Rfx6) gene. An Rfx6 knockout experiment in mice has further clarified the possible mechanism by which these mutations lead to the immediate onset of diabetes in newborns after birth. Although Rfx6 deletion does not affect the expression of Ngn3, a key transcription factor for endocrine differentiation during pancreatic development, the transcription factor RFX-6, a product of Rfx6 expression, directs islet cell differentiation in the downstream of Ngn3. Deletion of Rfx6 prevented the mice from generating islet cells other than PP cells, leading to their immediate manifestation of diabetes mellitus at birth due to the absence of β-cells [107, 108]. Heterozygous mutations in GATA binding protein 6 (GATA6) are also causes of NDM. GATA6 plays a crucial role in the development of human islets; hPSCs with heterozygous GATA6 mutations show reduced efficiency in differentiating into PP cells and β-like cells, leading to functional defects in glucose responsiveness [109].

## Challenges in construction and application of islet organoid

### Problems with extracellular matrix and 3D culture technology

#### Selection of matrix and medium

ECM and conditioned media with the addition of specific components constitute the ecological niche of stem cells and play a crucial role in stem cell self-renewal and differentiation. ECM used in organoid construction includes: Matrigel from Engelbreth-Holm-Swarm mouse sarcomas, natural polymers of biological origin, synthetic polymers, and decellularized ECM from human or animals. Matrigel derived from mice is the earliest and most widely used ECM for organoid culture, with the advantages of being easy to manipulate and containing a wide range of nutrients that support cell survival [110]. However, problems such as unknown composition, batch variability, and immunogenicity have restricted Matrigel’s clinical application, making the development of non-Matrigel matrices for organoid culture a focus of current research**(**Table 3**)**.Decellularized ECM from animals has the advantage of promoting the regeneration of damaged tissues, and its risk of immune rejection has been reduced by specific preparation techniques, thus entering clinical application. However, the impact of donor health on the survival rate of the inoculated cells and the development of organoids should not be ignored [111]. Natural polymers, including collagen, laminin, fibronectin, as well as alginate, hyaluronic acid, and chitosan, facilitate cell adhesion and cell-cell interactions. However, their biological origins also lead to defects such as batch variation and challenging mechanical property control. Polyvinyl alcohol (PVA), polycaprolactone (PCL), poly lactic-co-glycolic acid (PLGA), poly lactic acid (PLA), and polyethylene glycol (PEG) are common synthetic polymers that address natural polymers’ defects of batch variations and uncontrollable properties. Yet, the complex and expensive nature of their synthesis, alongside poor cell adhesion and bioactivity, and toxic byproducts from their degradation are unfavorable to organoid cultures [112]. Various synthetic hydrogels as new ECM alternatives address the challenges posed by synthetic polymers to cell growth. The Amikagel hydrogel system with well-defined composition allowed precise control over self-aggregation process to determine cell cluster sizes while generating islet organoids with enhanced maturity and GSIS [55]. However, poorly set up biochemical cues in the hydrogels can lead to the apoptosis of cells not adhered to the hydrogel, potentially explaining the limited duration of functional maintenance in organoids cultured within hydrogel systems [111]. Engineered recombinant gels can incorporate well-defined chemical cues, enabling controllable physicochemical properties and degradation rates. A recombinant protein gel system was developed by integrating the gene sequences of fibronectin (FN) and laminin (LAMA3) into polymerase enhancing peptide (PEP), which can support the growth of mouse EP cells and EC cells [111].

**Table 3:** The advantages and disadvantages of various matrixes and methods for islet organoids construction

Construction	Advantages	Disadvantages
Matrix		
Matrigel	Easy to operate, contains a variety of nutrients	Unknown composition and batch-to-batch variation, Immunogenicity
Decellularized ECM	Facilitates the regeneration of damaged tissue, Reduced risk of immune rejection through technical methods	Significant impact of donor condition on cell survival and development
Natural polymer	Better cell adhesion and intercellular interaction	Natural biological sources have batch variations and uncontrollable properties
Artificial polymer	Well-defined composition without batch-to-batch variation	Complex synthesis and expensive, Poor bioactivity not conducive to cell adhesion, Toxic byproducts produced by degradation
Engineered recombinant gel	Well-defined components, controllable physicochemical properties and degradation rate	Complex operation for inserting protein gene sequences
Method		
Suspension culture	Adequate signaling and interaction between cells and between cells and the matrix	Uneven size of spontaneously aggregated cell clusters
Scaffold culture	Controllable size and positioning of cell clusters	Potential immune rejection due to scaffold materials Thrombosis risk of scaffold transplants
Microfluidic chip culture	Better dynamic assessment of function, Better nutrient and oxygen support	Risk of microbial contamination in a well-supported nutritional environment Damage to cells from perfusion microbubbles

Adjustment of medium composition at different stages of cell differentiation and development is crucial for enhancing the maturation and function of islet organoids. A six-stage differentiation strategy was implemented using serum-free medium in contrast to previous approaches with serum medium, and it optimized the introduction of an ALK5 inhibitor, which inhibits TGF-β signaling, from stage 6 to stage 5, significantly enhancing cell differentiation and maturation and yielding a greater number of functionally superior β-cells [113]. Thereafter, an additional stage 7 (final maturation stage) was introduced by substituting the ALK5 inhibitor with the antiproliferative agent aurora kinase inhibitor ZM447439, which allowed the sc-islets to form a glucose-stimulated biphasic insulin secretion, showing high similarity to natural islets in terms of signaling transduction and exocytosis functions [114]. Besides the need for further optimization of the culture medium adjustment strategy, the existing strategies also require more reproducibility validation. This necessity arises from the possibility of intra- and inter-laboratory differences in culture media, which may be due to variations in the concentration of components such as growth factors, experienced by most laboratories while developing their customized conditioned media [115].

#### Challenges of 3D culture technology

3D culture techniques in the construction process of islet-like organoids include suspension cultures [13], cultures based on various types of scaffolds, and microfluidic chip cultures [115]. Advances in tissue engineering and 3D culture techniques have enabled rapid progress in generating organ tissues that resemble native islets in form and function. However, perfectly replicating the same physiological complexity remains challenging.

The commonly used suspension culture technique enables cells to spontaneously aggregate to form structures similar to human tissues, which is easy to operate and promotes signaling and interactions between cells and between cells and matrix. It was confirmed that β-cells derived from hESCs via suspension culture have the ultrastructural characteristics of human β-cells in vivo, and importantly, they are capable of insulin secretion in response to elevated glucose levels [116]. However, the cell clusters formed by spontaneous aggregation of hPSCs in suspension culture varied in size and had an average size larger than that of natural islets. Intriguingly, individual cells in smaller islets demonstrate a higher insulin secretion capacity compared to those in larger islets [31, 32]. Moreover, during the transplantation of cell clusters cultured in suspension, interactions between cells and between cells and the matrix may be compromised. Microporous scaffold culture technology, as a more optimal alternative, can be control the size of aggregated cell clusters and cell-cell and cell-matrix interactions through the diameter of the microwells, thus obtaining islet organoids of uniform size, with a preserved signaling environment and insulin-producing response to glucose [117].

Despite these advantages over suspension culture techniques, scaffold culture techniques based on various materials also face multiple challenges. Firstly, the selection of scaffold materials requires further refinement, as natural and synthetic scaffolds each have their own advantages and disadvantages, and the choice of materials to be used individually or in combination needs to be specifically adapted to the cultured cells [118]. Even though PLG and PEG bioscaffolds are similar in structure and other designs, only cells cultured on PLG scaffolds demonstrated higher expression of cellular adhesion factors compared to suspension cultures, while cells cultured on PEG scaffolds exhibited a significant increase in the expression of β-cell maturation marker genes [117]. Another challenge is the difficulty of seeding cells into the scaffolds, which affects the precise positioning and proliferation of cells. The use of 3D bioprinting technology realizes the creation of scaffolds with precise cellular localization and structural distribution [119]. Furthermore, the bio-inks of specific compositions offer a supportive environment more favorable for cell survival, providing a powerful technology support for scaffold-based culture of islets for diabetes transplantation therapies [120]. However, although current advancements in 3D bioprinting technology enabling the promotion of vascular formation in scaffolds by incorporating ECs and vascular endothelial growth factors, the fragility of the formed vessels restricts the success rate of transplantation, necessitating the exploration of new strategies for improvement [121].

Compared with the static culture system, the microfluidic dynamic culture system provides an enhanced nutrient-supportive environment for the cells, resulting in reduced hypoxic stress in the generated islet organoids, elevated insulin secretion function, and a much longer time of organ viability and effective function [122]. However, experimental results have also shown that air bubbles produced by the continuous perfusion in the microfluidics system adversely affect chip control and can harm cells. Moreover, the enhanced nutrient support environment also increases the risk of microbial contamination [85].

### Differences between islet organoids and natural islets

**Fig. 3:**
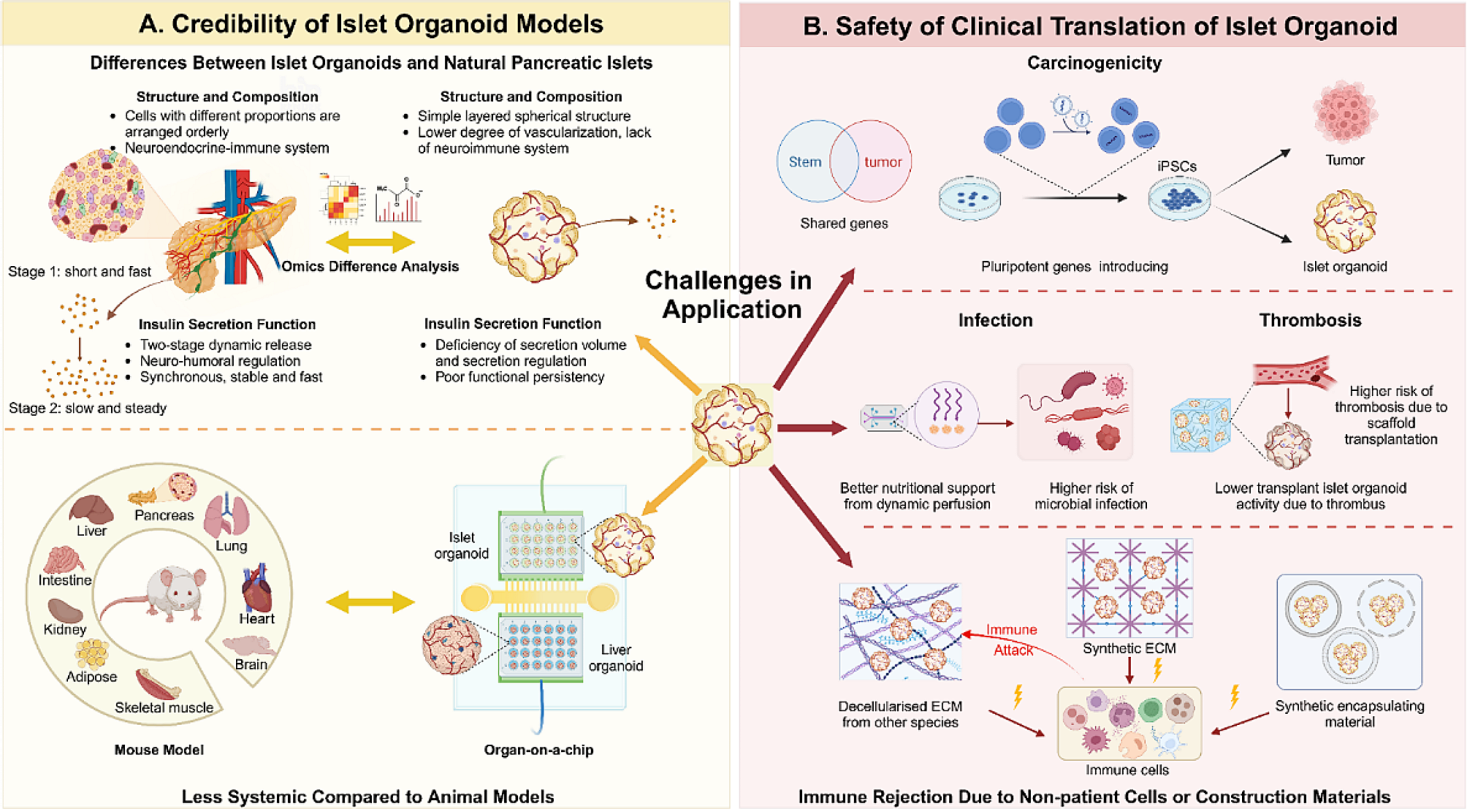
Challenges in application of islet organoid regarding model credibility and translation safety. **A. Credibility of islet organoid models.** Islet organoids exhibit significant differences from natural islets in: overall structure, cellular composition, functional regulation, transcriptomes, and metabolomes. This diminishes the credibility of researches based on islet organoid models **(top left)**. Meanwhile, for the study of diseases like diabetes, whose onset and progression involves multiple systems, organoid chip systems that lack integrated organs remain inferior to model organisms such as mice **(bottom left)**. **B. Safety of clinical translation of islet organoid.** Currently, there are no clinical studies on the transplantation of islet organoids into humans, and the unclear safety of transplantation is a significant concern. Potential risks include: tumorigenicity of iPSCs with high proliferation and differentiation potential **(top right)**; high microbial contamination potential of well-nourished dynamic culture systems and thrombosis susceptibility of 3D scaffold transplants **(middle right)**; and while patient-derived or encapsulation measures have mitigated immune rejection damage, the materials used for the construction and encapsulation of islet organoids may also activate the immune attack **(bottom right)**

Although islet organoids constructed currently are capable of insulin secretion in response to glucose stimulation, they still exhibit a few functional deficiencies compared to natural islets, significantly affecting the exploration of the mechanisms behind these functional abnormalities and the development or improvement for functional restoration therapies. The functional defects of individual sc-β cell are characterized by lower secretion levels and deficiencies in release regulation compared with β-cells in vivo. For example, under high glucose stimulation, the insulin release from sc-β cells lacks the biphasic dynamic release process, which should consist of an initial phase of brief rapid release followed by a secondary phase of sustained slow release [113]. An important reason for the functional defects of individual sc-β cells is that their maturity levels are lower than that of β-cell in vivo. β-cells with higher maturity level have increased insulin secretion and can selectively respond to blood glucose levels that exceed normal physiologic concentrations. Consequently, further studies a is essential to determine the effects of various factors, including nutrients, growth factors, oxygen content, mechanical stress, on cell maturation, which will aid in identifying strategies to enhance the maturity of cells cultured in vitro [123] **(**Fig. 3, **Created with BioRender.com)**.

In addition, the overall function of islet organoids is also defective compared to that of natural islets, such as the lack of an intact neurohumoral regulatory system and a synchronized, stable, and rapid insulin release from numerous β-cells based on it [124]. The defects in the overall function of islet organoids may also be associated with factors like the vascularization levels, the composition and arrangement of EC cells, and the heterogeneity of β-cells. Insufficient vascularization hampers β-cell maturation, severely compromising its function : hypoxic stress and nutrient deficiencies can lead to cell apoptosis and hypoplasia, while inhibited intercellular signaling between vascular and β-cells interferes with the normal regulation of metabolic waste removal and humoral response [125]. Most islet organoids consist of primarily β-cells, with α, β, δ, and auxiliary cells making up only a minor fraction. Additionally, these cells are often merely arranged in spherical layers, lacking the intricate microarchitecture of natural islets, where diverse cell types are meticulously organized [112], while the proportion and arrangement of EC cells have been shown to be crucial for islet function [126]. Besides, natural pancreatic islets have a high level of β-cell heterogeneity, which is essential for effective and diverse blood glucose regulation. Islet organoids, by contrast, show a pronounced deficit in β-cell heterogeneity due to the absence of cell polarity and the interactions among vascular, neural, cellular, and microenvironmental components, which limits their capacity for effective, holistic, and varied blood glucose regulation [127].

Islet organoids also exhibit deficiencies in the onset and duration of their function. hPSC-derived islets, despite significant improvement in cell maturity, released insulin in response to glucose stimulation only after 2–6 weeks after transplantation into the mice, different from human islets that function immediately after transplantation [128]. The inability of sc-β cells to immediately regulate blood glucose levels after transplantation may result from graft remodeling that occurs after transplantation, including progenitor cell differentiation and maturation enhancement, suggesting a need for further improvement of current strategies for induced differentiation and culture of organoids [24]. On the other hand, it was found that hPSC-derived β-cells cultured in vitro maintained their function after transplantation into diabetic mice only for a short period of time, and that the declining blood glucose levels of mice rebounded again over time [116]. Inadequate maintenance of function after transplantation may be linked to mutations in source cells, for example, β-cells with heterozygous hepatocyte nuclear factor 1 alpha (HNF1A) mutation, which causes MODY, do not directly show a lack of insulin secretion, but rather a gradual loss of insulin secretion [129]. Cell loss owing to insufficient vascularization and immune rejection could also be possible causes.

Besides function defects, there are still several differences between isle organoids and natural pancreatic islets, which may affect the reliability of the research findings on related disease. In terms of developmental stage, the β-cell transcriptome in hPSC-derived islet organoids closely resemble those of fetal β-cells. While this similarity benefits research into diabetes linked with abnormal islet development, it somewhat weakens the translational ability of studies on the pathogenesis and treatment of diabetes that progresses later in life [130]. Beyond the transcriptomic differences, islet organoids and natural pancreatic islets also vary in their metabolomic profiles. The extent to which differences in metabolism, such as glycolysis and mitochondrial glucose metabolism, affect the results of studies such as drug screening for diabetes treatment warrants further investigation. Moreover, although the pathogenesis of diabetes is closely related to abnormalities in β-cell number and function, islet organoids, predominantly composed of β-cells, lack non-pancreatic components such as blood vessels, neural networks, and immune cells, which play crucial roles in β-cell survival, development, and function. They also differ significantly from natural pancreatic islets in terms of morphology, structure, and size [131].

### Safety of islet organoid transplantation therapy

#### Tumorigenicity

Current protocols for directed differentiation of stem cells generate heterogeneous proliferating cells that are not present in natural islets, posing a potential risk for tumor formation after transplantation [132, 133]. Similarly, previous research has demonstrated that iPSCs derived from different adult mouse tissues can evolve into teratomas and vary widely in their tendencies to form such tumors [134]. Induction of PSCs by cellular myelocytomatosis oncogene (c-Myc) gene transduction is a strategy known to increase the risk of tumorigenicity. The product, the transcription factor c-MYC, is crucial in regulating cell proliferation, differentiation, and development. While it markedly boosts iPSC generation, it also leads to about 20% of progeny cells evolving into tumors [135].

In addition to avoiding source tissues with high tumorigenic tendencies and induction strategies that incorporate high tumorigenic risks, defining more precise cell surface markers for cell sorting and using antibody-mediated selective elimination of tumorigenic cells, as well as developing selective chemical inhibitors and drugs that regulate suicide gene cassettes, are effective strategies to enhance the safety of β-cells derived from PSC [136].

#### Immune rejection

Although the construction of islet organoids derived from patient stem cell effectively solves the problem of transplant immune rejection, the broad application of clinical-grade autotransplants may be limited by factors such as cost [20], allogeneic transplantation remains the primary approach, and the corresponding transplant rejection is a concern during organoid construction.

Immune rejection reactions caused by allogeneic islet transplantation include hyperacute rejection, acute rejection, and chronic rejection, with acute rejection being the most common [137]. HLA is a major cause of transplant rejection. Antigen-presenting cells (APCs) of the recipient recognize the donor cell’s allogeneic HLA, process it into an exogenous peptide-self HLA complex, and present it on the cell surface, which is then recognized by the recipient’s T-cell receptor (TCR), activating T cells and leading to transplant rejection. Therefore, gene editing of transplant cells to selectively delete HLA molecules is an effective strategy to inhibit rejection. For example, β-cells derived from genetically engineered hPSCs with deleted class I HLA molecules significantly suppress T-cell activation [138]. However, deficiencies in HLA molecules can also increase the risk of malignancy and infection in the transplant cells and downregulation of HLA expression may activate natural killer cells (NK cells) [139]. Reducing macrophage phagocytosis of presented allogeneic HLA and related antigens, such as by introducing the cluster of differentiation 47 (CD47) molecule (‘self ' marker on normal cells) into transplant cells via lentiviral vectors to interact with signal-regulatory protein alpha (SIRPα), has successfully enhanced the survival rate of xenogeneic transplant cells [140]. Notably, overexpression of CD47, despite its definite immunosuppressive effect, inhibits insulin secretion by activating the cell division control protein 42 (Cdc42), thus affecting the efficacy of the transplant [141]. Adaptive immune responses by T cells plays a crucial role in the occurrence of immune rejection, while the interaction between the immune checkpoint programmed death-ligand 1 (PD-L1) and the programmed cell death protein 1 (PD-1) ligand on T cell surfaces can suppress the effector functions of T cells. Applying interferon-gamma (IFN-γ) to stimulate PD-L1 overexpression in islet organoids effectively protected xenogeneic islet organoids transplanted in immunocompetent diabetic mice, while maintaining effective glucose reduction for up to 50 days [142]. However, mere overexpression of PD-L1 is insufficient to overcome immune rejection. It requires increasing the expression of interleukin-10 (IL-10) and TGF-β through plasmid-mediated gene delivery to induce the generation of regulatory T cells (Tregs) [143], or introducing other immunosuppressive proteins such as indoleamine 2,3-dioxygenase (IDO) via lentiviral transduction to enhance immune protection [144]. These in vitro experiments and xenogeneic transplantation models to some extent confirm that gene editing and engineering may effectively suppress immune rejection responses. However, the lack of a complete immune system assessment (such as experiments involving only T cells without evaluating the impact of NK cells) and the effects of xenogeneic binding affinity of immune checkpoint molecules and their ligands limit the extension of these conclusions to allogeneic transplantation. Moreover, the timing and mechanisms of these suppression strategies still primarily correspond to those of immunosuppressive treatments that are able to ameliorate acute rejection responses, but they do not fully resolve the issue of chronic rejection reactions that occur occurs in the later stage after transplantation. There is also a potential increase in the risk of malignancy and infection, thus the practical application of these strategies requires further improvement and comprehensive evaluation.

At the same time, biologically derived or synthetically produced matrix and scaffold materials also exhibit some degree of immunogenicity [145]. Encapsulating islets within a physical semipermeable barrier constructed from specific materials is also a feasible strategy, but encapsulated islets may have problems such as the inability to maintain secretion function. Further improvement of the encapsulation barrier is needed, for instance, reducing its thickness to prevent immune cell infiltration while maximizing the free diffusion of nutrients, insulin, glucose, and other small molecular substances, thereby improving its glucose regulation capabilities [146]. Moreover, the novel encapsulation materials themselves might induce immune rejection due to their foreign origin [147, 148].Therefore, ongoing research and development are focused on strategies to suppress immune rejection, including organoid immune modification and biomaterial encapsulation.

#### Infection and thrombosis

Biologically sourced media lacking quality control, such as fetal bovine serum, are potential sources of infection due to their pathogen content [149]. Additionally, microfluidic systems that provide better nutritional support to cells through continuous perfusion also face challenges with microbial contamination [85]. Presently, islet organoids constructed do not possess a functional immune system, thereby amplifying their susceptibility to infections from such contaminations [150].

In addition to insufficient vascularization and endothelial function defects [125], organoid culture scaffolds are prone to thrombosis, which would severely reduce the viability of transplanted organoids [151]. This may be due to poor anticoagulation and biomechanical properties of the scaffolds, as well as the exposure to scaffold collagen promoting coagulation after reperfusion. In view of this problem, the heparinized-vascular endothelial growth factor-defoliated kidney scaffolds (HEP-VEGF-DKSs) effectively reduced platelet adhesion and enhanced neovascularization capabilities, suggesting a potential strategy that could be applied to scaffolds for islet organoids culture, though this application remains to be validated [152].

## Outlook

Islet organoid technology has made significant progress in recent years, but constructing islet organoids is a complex process. Firstly, the cell types in currently cultured islet organoids are relatively simple, only simulating part of the pancreatic physiological functions. Therefore, one of the challenges is to adjust and optimize existing culture conditions, which will help develop more functionally mature islet organoids that better replicate the complex biological functions of the islets. Given the differences between natural islets and islet organoids, multi-omics approaches can be employed to identify molecules influencing the construction of islet organoid and then improve its function at the genetic level by gene editing technologies. Secondly,. complex communication and regulatory mechanisms exist among different cell types of the pancreas, but current islet organoids have not yet developed a complete and compatible interactive co-culture system. Currently, the co-culture of islet organoids is a hot topic of research, islet is a heterogeneous organ, and a non-negligible drawback of organoid culture is the lack of blood vessels and immune cells, thus co-culture strategies should be explored based on current culture protocols.

The application of islet organoids in diabetes mellitus has seen considerable advancements. In clinical treatment, islet organoids are mostly used for T1DM, characterized by an absolute deficiency of insulin. The common clinical strategy for islet cell transplantation is through the portal vein of the liver. However, transplantation within the hepatic portal vein carries a high risk of bleeding and coagulation, and an immediate inflammatory response often occurs in the early stages of islet transplantation, leading to substantial islet cell death. A novel transplantation strategy is the sub-anterior rectus sheath transplantation. This surgical approach is safe and easy to perform, involving no risks of bleeding or coagulation during the process, effectively supporting the early cell survival and long-term function maintenance of the islet organoids [21]. For other subtypes such as T2DM, drug therapy to lower blood sugar levels is still considered first, and the use of islet organoids remains limited. Current studies have used islet organoids in patients with advanced and severe T2DM that cannot be controlled by drugs, suggesting that the clinical indications for islet organoids could be further expanded in the future. Additionally, the existing islet organoid culture systems are relatively complex and have long differentiation cycles, which significantly hinder clinical translation. Developing ‘universal islets’ as off-the-shelf products is a direction to improve product accessibility, and such ‘universal islets’ offer diabetes treatment without the need for immunosuppression. Understanding the pathogenesis of diabetes through studies on the proteome and metabolome of islet cells could identify key molecules and metabolic pathways associated with diabetes, providing new targets for early diagnosis and intervention. Moreover, gene editing can be applied to islet organoids to study the regulatory mechanisms of insulin secretion and explore the pathological changes in islet cells in diabetes. For drug screening, islet organoids are used as high-throughput screening models. They demonstrate the ability to rapidly identify drugs with strong sensitivity and superior effects in screening large compound libraries and assessing drug concentrations for anti-diabetic medications. For diabetes, a systemic disease affecting multiple organs and systems, the interactions between organs and systems play a crucial role in disease progression, yet current therapeutic and research efforts involving islet-like organoids only address single organ systems. Constructing multi-organ interconnected organoid systems, such as gut-pancreas organoid systems [82], and integrated liver-gallbladder-pancreas organ chips to consolidate all organs related to diabetes metabolism [81], are viable strategies to overcome the limitations of single organ studies. As cells from different organs require optimal culture components and timelines, multi-organ chips require further improvements in culture and construction schemes to determine the optimal conditions that support long-term cultivation of all organs involved.

For the construction of islet organoids, it is necessary to closely mimic the structure and function of the pancreatic organ to establish the optimal model for diabetes research. Although advances in tissue engineering and 3D culture techniques have made significant strides in creating organ tissues similar in morphology and function to native islets, replicating the same physiological complexity perfectly remains challenging. Future technological developments will also provide more flexible growth devices, suitable porous formats for screening, and microfluidic systems. Microfluidic systems will be able to retrieve multiple physiological parameters in parallel and connect organoids models of different human organs, thereby reproducing inter-organ communication and constructing a multi-organ composite organoid system. Bioengineering and bioprinting technologies are also continuously evolving to provide devices that improve organ structure. Regarding the safety of treatments using islet organoids, the potential risk of tumorigenesis is a significant concern, which might hinder the development of therapeutic strategies based on islet organoids. If undifferentiated hESC and hiPSC remain in the final islet organoids, there is a clinical risk of forming malignant teratomas upon implantation. To reduce the risks of off-target differentiation and tumorigenicity, fluorescence or magnetic activated cell sorting techniques can be used to select differentiated cells based on specific PP cell or β cell markers. Additionally, undifferentiated hPSCs can be removed from the final islet cell clusters through treatment with antibodies and small molecules. Immune rejection is another issue faced by islet organoids. To overcome this, encapsulation devices have been developed to protect the organoid models from the immune system. Furthermore, gene editing of islet organoids or the combined use of immunosuppressive agents are effective strategies to suppress immune rejection.

In summary, islet organoids are considered an unlimited organ resource for transplantation and treatment of diabetes, as well as a model for studying diabetes mechanisms that can simulate the human body environment. Although several issues and safety concerns regarding organoids transplantation remain to be resolved, efforts to optimize organ transplantation techniques for islet organoids continue to evolve. The development of islet organoids will be a milestone in understanding and treating diabetes.

## Data Availability

Not applicable.

## Abbreviations

ASCs: Adult Stem Cells
ADSCs: Adipose Tissue Stem Cells
ALK: Anaplastic Lymphoma Kinase
Arx: Aristaless-Related Homeobox
AAVS1: Adeno-Associated Virus Integration Site 1
ALK5: Activin Receptor-Like Kinase 5
BMP: Bone Morphogenetic Protein
BMSCs: Bone Marrow Mesenchymal Stem Cells
CDK9: Cyclin-Dependent Kinase 9
CDKAL1: CDK5 Regulatory Subunit Associated Protein 1-Like 1
CRISPR/Cas9: Clustered Regularly Interspaced Short Palindromic Repeats-Associated Protein 9
JNK: c-Jun N-terminal Kinase
CFTR: Cystic Fibrosis Transmembrane Conductance Regulator
CF: Cystic Fibrosis
CpG: Cytosine-Phosphate-Guanine
c-Myc: Cellular Myelocytomatosis Oncogene
CD47: Cluster of Differentiation 47
Cdc42: Cell Division Control Protein 42
DE: Definitive Endoderm
DYRK1A: Dual-Specificity Tyrosine Phosphorylation Regulated Kinase 1 A
Dnmt1: DNA Methyltransferase 1
EP: Endocrine Precursor Cells
EP Cells: Endodermal Progenitor Cells
ESCs: Embryonic Stem Cells
EnSCs: Endoderm Stem Cells
E-islets: EnSCs Based Islets
EC cells: Endocrine Cells
ECs: Endothelial Cells
EGF: Epidermal Growth Factor
ECM: Extracellular Matrix
EBs: Embryoid Bodies
ER: Endoplasmic Reticulum
EEC: Enteroendocrine Cells
FGF2: Fibroblast Growth Factor 2
FOXOs: Forkhead Box O Transcription Factors
FOS: FBJ Murine Osteosarcoma Viral Oncogene Homolog
FN: Fibronectin
GCK: Glucokinase
GSIS: Glucose-Stimulated Insulin Secretion
GWAS: Genome-Wide Association Study
GLIS3: GLI-Similar 3
GLP-1: Glucagon-Like Peptide-1
GATA6: GATA Binding Protein 6
GATA4: GATA Binding Protein 4
GMP: Good Manufacturing Practices
HEP-VEGF-DKSs: Heparinized-Vascular Endothelial Growth Factor Defoliated Kidney Scaffolds
HNF3B: Hepatocyte Nuclear Factor 3 Beta
HGF: Hepatocyte Growth Factor
HB9: Homeobox Protein HB9
hAECs: Human Amniotic Epithelial Cells
hESCs: Human Embryonic Stem Cells
hPSCs: Human Pluripotent Stem Cells
hiPSCs: Human Induced Pluripotent Stem Cells
HLSC: Human Liver Stem Cells
hGSCs: Human gastrointestinal Stem Cells
HUVECs: Human Umbilical Vein Endothelial Cells
HNF1A: Hepatocyte Nuclear Factor 1 Alpha
HLA: Human Leukocyte Antigen
HNF1αp291fsinsC: Hepatocyte Nuclear Factor 1 Alpha p291 Frameshift Insertion C
HNF1α WT iPSC: Hepatocyte Nuclear Factor 1 Alpha Wild-Type Induced Pluripotent Stem Cells
HNF1β: Hepatocyte Nuclear Factor 1 Beta
IUGR: Intrauterine Growth Retardation
IDO: Indoleamine 2,3-Dioxygenase
IFN-γ: Interferon-Gamma
IL-10: Interleukin-10
ITS: Insulin-Transferrin-Selenium
ICs: Isolated Islet Cells
IFNα-Induced Antigens: Interferon Alpha-Induced Antigens
iPSCs: Induced Pluripotent Stem Cells
JUN: Jun Proto-Oncogene
JAZF1: Juxtaposed with Another Zinc Finger Gene 1
LIN28B: Lin-28 Homolog B
LIF: Leukemia Inhibitory Factor
LAMA3: Laminin
MSCs: Mesenchymal Stromal Cells
NDM: Neonatal Diabetes Mellitus
NEUROD1: Neuronal Differentiation 1
NGN3: Neurogenin3
Ngn3ER: NGN3 and Estrogen Receptor
NG2: Neuron-glial antigen 2
NKX2.2: NK2 Homeobox 2
NKX6.1: NK6 Homeobox 1
PBMCs: Peripheral Blood Mononuclear Cells
PSCs: Pluripotent Stem Cells
PDX1: Pancreatic and Duodenal Homeobox 1
PP cells: Pancreatic Progenitor Cells
PDX: Patient-Derived Xenograft
PCL: Polycaprolactone
PDMS: Polydimethylsiloxane 1
PVA: Polyvinyl Alcohol
PEP: Polymerase Enhancing Peptide
PEG: Polyethylene Glycol
PLGA: Poly Lactic-Co-Glycolic Acid
PD-1: Programmed Cell Death Protein 1
PD-L1: Programmed Death-Ligand 1
PLA: Poly Lactic Acid
REST: RE1-Silencing Transcription Factor
ROS: Reactive Oxygen Species
RFX6: Regulatory Factor X 6
APCs: Antigen-Presenting Cells
SIRPα: Signal-Regulatory Protein Alpha
SNP: Single-Nucleotide Polymorphism
Shh: Sonic Hedgehog
sc-β Cells: Stem-Cell-Derived β-Cells
sc-islets: Stem-Cell-Derived β-Cells
STAT3: Signal Transducer and Activator of Transcription 3
2D: Two-Dimensional
3D: Three-Dimensional
T1DM: Type 1 Diabetes Mellitus
T2DM: Type 2 Diabetes Mellitus
T3: Triiodothyronine
TYK2: Tyrosine Kinase 2
TALEN: Transcription Activator-Like Effector Nuclease
TCF7L2: Transcription Factor 7 Like 2
TGF-β: Transforming Growth Factor Beta
T Cells: T Lymphocytes
TCR: T Cell Receptor
MAFA: V-MAF Avian Musculoaponeurotic Fibrosarcoma Oncogene Homolog A
MAFB: V-MAF Avian Musculoaponeurotic Fibrosarcoma Oncogene Homolog B
VE-cadherin: Vascular Endothelial Cadherin
WNT: Wingless/Integrated
WNT4: Wingless-Type MMTV Integration Site Family, Member 4
WFS: Wolfram Syndrome
YAP: Yes-Associated Protein
ZFN: Zinc Finger Nuclease
